# The impact of protein quality on the promotion of resistance exercise-induced changes in muscle mass

**DOI:** 10.1186/s12986-016-0124-8

**Published:** 2016-09-29

**Authors:** Stuart M. Phillips

**Affiliations:** Department of Kinesiology, McMaster University, 1280 Main St., West Hamilton, ON L8S 4K1 Canada

**Keywords:** Leucine, Hypertrophy, DIAAS

## Abstract

Protein supplementation during resistance exercise training augments hypertrophic gains. Protein ingestion and the resultant hyperaminoacidemia provides the building blocks (indispensable amino acids – IAA) for, and also triggers an increase in, muscle protein synthesis (MPS), suppression of muscle protein breakdown (MPB), and net positive protein balance (i.e., MPS > MPB). The key amino acid triggering the rise in MPS is leucine, which stimulates the mechanistic target of rapamycin complex-1, a key signalling protein, and triggers a rise in MPS. As such, ingested proteins with a high leucine content would be advantageous in triggering a rise in MPS. Thus, protein quality (reflected in IAA content and protein digestibility) has an impact on changes in MPS and could ultimately affect skeletal muscle mass. Protein quality has been measured by the protein digestibility-corrected amino acid score (PDCAAS); however, the digestible indispensable amino acid score (DIAAS) has been recommended as a better method for protein quality scoring. Under DIAAS there is the recognition that amino acids are individual nutrients and that protein quality is contingent on IAA content and ileal (as opposed to fecal) digestibility. Differences in protein quality may have important ramifications for exercise-induced changes in muscle mass gains made with resistance exercise as well as muscle remodelling. Thus, the purpose of this review is a critical appraisal of studies examining the effects of protein quality in supplementation on changes in muscle mass and strength as well as body composition during resistance training.

## Background

Performing resistance exercise results in a fundamentally anabolic stimulus for skeletal muscle. When performed often enough resistance training can result in varying degrees of muscular hypertrophy [1]. The degree of hypertrophy seen with resistance exercise training is, however, highly variable [2–7] and likely dependent on a number of variables including a persons’ prior training status, sex, and age; however, the purpose of this review is not to interrogate these variables and the interested reader is directed to the following reviews [8–10]. A number of lines of evidence show that ingestion of supplemental protein during a program of resistance training can augment the hypertrophic and strength responses [11]. Research has been undertaken to try and define how protein can augment resistance training-induced training responses and relevant variables have been examined including protein quantity (dose) and protein timing (relative to exercise). While beyond the scope of this review, recent reviews looking at the issues of protein dose [8] and protein timing [12] are also excellent adjuncts to the topic covered here. The impact of protein quality, using PDCAAS estimates, on skeletal muscle anabolic responses, at rest, have recently been comprehensively reviewed [13]; however, there has been less attention paid to the impact of protein quality as a variable and its importance in driving resistance training-induced hypertrophy. This is despite several instances showing that protein quality can affect the acute post-exercise muscle protein synthetic response [14] and muscle hypertrophy with resistance training [5, 15, 16]. The focus of this review is solely on muscular hypertrophy as a result of resistance training and protein supplementation and not on strength. Recognizing that strength gains as a result of resistance training, which while dependent on gains in muscle mass/cross-sectional area, are likely equally as dependent (or possibly even more so) on gains in neuromuscular factors. The aim of this review is to examine how lower versus higher quality proteins impact the adaptations to resistance exercise training with a focus on skeletal muscle hypertrophy.

### The role of supplemental protein in promoting muscle hypertrophy

Muscle hypertrophy following resistance training is the result of several processes [17] that include changes in satellite cell content and activity (for review see [18]), as well as protein turnover (for review see [8]). It is clear from a number of studies that resistance exercise ‘sensitizes’ the muscle to hyperaminoacidemia [19]. Thus, resistance exercise has the effect of acting in a synergistic manner with the normal rise in muscle protein synthesis (MPS) that occurs with protein feeding (see the following reviews [8, 20, 21] for more detail). Ultimately, resistance exercise results in periods of extended positive muscle protein balance, greater than those with feeding alone. The effect of these extended periods of positive protein balance is that the muscle fiber undergoes addition of contractile protein mass and increases the fiber size [8]. Supplementation (i.e., consumption of protein over and above a habitual protein intake) of a persons’ normal dietary intake with various protein sources has been shown to augment the hypertrophic response with resistance training in both younger and older participants [11, 22]. A meta-analysis shows that protein timing (i.e., rapid consumption within a certain time period pre-, during, or post-exercise) is not as important in determining strength or hypertrophic gains [12]. Nonetheless, a pragmatic recommendation for athletes and resistance trainees would still be to begin recovery from exercise as soon as possible. Thus, post-exercise protein consumption (as well as hydration and carbohydrate provision) has been shown to be effective at stimulating MPS (reviewed here and elsewhere [8, 21]) and thus is recommended over pre-exercise [23–25] or during-exercise provision of protein [26], which would have a more variable effect on MPS and possibly resistance training-induced hypertrophy.

It has been shown that only the indispensable amino acids (IAA) are required for the stimulation of MPS [27–29] (here the term indispensable amino acid, as opposed to essential amino acid is used in keeping with recommended usage [30, 31]). Of the IAA a position of prominence belongs to leucine as an amino acid that acts as a signaling molecule to stimulate MPS, as well as being a building block for protein [32–35]. The mediation of MPS by leucine is through the mechanistic target of rapamycin complex-1 (mTORC1; for reviews see [36–38]). It has recently been shown that a protein named Sestrin2 is the leucine-binding sensor for mTORC1 [39, 40]. Upon leucine binding with Sestrin2 there is dissociation of Sestrin2 and GATOR2 (a GTPase-activating protein) and activation of mTORC1 to allow it to phosphorylate/de-phosphorylate downstream proteins and activate MPS. Thus, when leucine binds to Sestrin2 this would stimulate MPS. The result, as hypothesized [41–43], is that leucinemia (and subsequently intracellular leucine concentration) following protein ingestion is a more likely determinant of muscle protein accretion than total protein alone. With these new understandings of how leucine is a stimulator of MPS, it is noteworthy that commonly consumed sources of supplemental proteins vary greatly in their leucine content. A brief discussion of protein quality is important at this stage to understand the potential for how not only protein quantity, but protein quality can impact changes in MPS and potentially muscle mass with resistance training.

### Protein quality: PDCAAS and DIAAS

Typically, protein quality has been measured using the protein digestibility-corrected amino acid score or PDCAAS [44–46]. More recently, a new, ostensibly superior, method for estimating protein quality has been proposed called the digestible indispensable amino acid score or DIAAS [31]. The main reasons for the derivation of the newer method of scoring protein quality related to concerns around the use of PDCAAS and an increasing recognition that digestibility could not be estimated from feces. Some of the bases for why DIAAS is suggested to be superior to PDCAAS are reviewed briefly here. First, dietary amino acids should be treated as individual nutrients and not simply as protein. Second, it was acknowledged that any digestibility correction had to recognize the difference between fecal, as opposed to ileal, protein digestibility. This is because there is a rapid, and often times substantial metabolism in terms of proteins, amino acids, and nitrogen in the colon. Thus, ileal (i.e., end intestinal) rather than fecal (which would include substantial microbiotic metabolism) digestibility is considered to be more accurate due to colonic protein and nitrogen metabolism [31]. Third, there was a growing recognition that certain amino acids, lysine as an example, can be reduced in terms of their bioavailability due to chemical reactions that can decrease the digestible lysine [31, 47]. Finally, there was concern raised over truncation of the PDCAAS value, which implied that amino acids available in terms of their content over and above those contained in a reference protein, where not useful. The point was made that truncation removes any nutritional differences between higher IAA-containing foods even if certain IAA may be limiting in some diets/proteins. Thus, the solution is to allow proteins, and the diets they are part of, to have an amino acid score of ≥ 1.0. Table 1. Shows the PDCAAS scores of some proteins and their corresponding DIAAS score as well as the limiting amino acid. The equation used to calculate the DIAAS, more details for which can be found here [31], is given below:

**Table 1:** PDCAAS and DIAAS scores, the limiting amino acid assessed by the amino acid reference ratio for selected proteins. Values from [47]

Protein source	PDCAAS	DIAAS	Limiting AA
Whey PI	1.00	1.09	His
Whey PC	1.00	0.97	His
Soy PI A	1.00	0.91	Met + Cys
Soy PI B	0.98	0.90	Met + Cys
Pea P	0.89	0.82	Met + Cys
Rice PC	0.42	0.37	Lys

*PDCAAS* protein digestibility-corrected amino acid score, *DIAAS* digestible indispensable amino acid score, *AA* amino acid, *PC* protein concentrate, *PI* protein isolate. Values are from reference [47]. Whey protein isolate (Whey PI) 8855, and whey protein concentrate (Whey PC) 392: all from the Fonterra Co-operative Group; soy PI A (Supro 670) and soy PI B (Supro XF) were from Solae; pea PC (Nutralys S85) from Roquette; and rice PC (Oryzatein 90) was from Axiom Foods. Limiting AA – first limiting amino acid when compared to an ideal protein

### DIAAS = (mg of digestible dietary IAA in dietary test protein)/(mg of the same IAA in the reference protein)

Importantly, the reference protein in this equation is not egg (as it was for PDCAAS) but a theoretical protein that covers all of the known requirements for the IAA. However, as with protein and the RDA, the requirement levels of IAA that form the DIAAS score reflect the minimum levels of intake of each amino acid. Thus, there is no attempt to define ‘optimal’ and potentially unique roles of amino acids such as leucine, as a stimulator of MPS, as well as its role as a substrate for the same process.

**Fig. 1:**
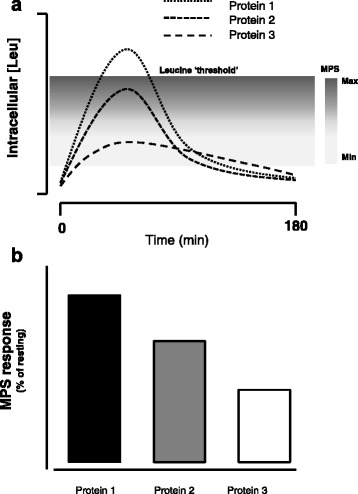
Schematic showing the impact of proteins with differing leucine contents on skeletal muscle fibre intracellular concentration of leucine and subsequent stimulation of muscle protein synthesis (MPS). The concept is that certain proteins with high leucine content and digestibility would raise intracellular Leu concentration and bind with Sestrin2 (see text for details) to trigger activation of MPS of varying degrees (**a**). The resultant MPS response (shown as a percent stimulation above resting) is shown in (**b**)

**Fig. 2:**
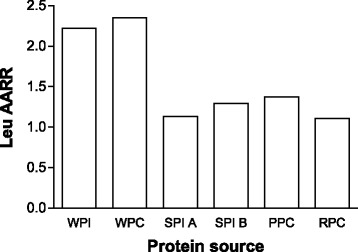
The Leucine (Leu) amino acid reference ratio (AARR – defined as the content of leucine in the protein measured compared to a hypothetical best protein to provide the EAA needed) for various protein concentrates and protein isolates. Values are from reference [47]. Whey PI and PC 392: all from the Fonterra Co-operative Group; soy PI A (Supro 670) and soy PI B (Supro XF) were from Solae; pea PC (Nutralys S85) from Roquette; and rice PC (Oryzatein 90) was from Axiom Foods. Values are from reference [47]

Beyond merely acting as a substrate, leucine has been shown in a number of studies to be the AA that turns on MPS [32–35]. This idea has been termed the leucine ‘threshold’ [8, 9, 41, 48], which is essentially a thesis that states that a rapid (i.e., from easily digestible proteins) post-prandial leucinemia, and likely the subsequent intracellular leucine concentration (presumably through leucine binding to Sestrin2 [39]), that triggers a rise in MPS (Fig. 1). Thus, in situations where MPS is the variable to be maximized it would be more important to focus on the available leucine content (if known), rather than a total IAA content. Thus, the leucine threshold is an important concept since leucine is the amino acid that turns on MPS [41, 48, 49] and, importantly, is most likely the critical amino acid that is an important driver of muscle hypertrophy. This thesis forms the basis of examination of studies in which proteins have been compared with respect to not only their ability to stimulate MPS, which is arguably a short-term measure not in complete agreement with longer-term resistance training outcomes [6, 50], but to promote resistance exercise-induced gains in muscle mass. Protein amino acid content, which when referenced to the ideal protein used in calculating DIAAS, yields the amino acid reference ratio (AARR). The AARR for leucine is shown for seven commonly consumed supplemental proteins (Fig. 2). What is important to recognize is that leucine is not, however, considered the limiting amino acid (i.e., the amino acid present in lowest abundance), which are shown in Table 1 for the same proteins.

In relation to the leucine trigger thesis (Fig. 1), it appears that the elderly have a greater leucine threshold and thus require greater levels of protein/leucine to stimulate MPS both at rest [42, 51] and following resistance exercise [52, 53]. Thus, in an effort to produce greater gains in muscle mass in the elderly, both with protein supplementation in the absence of exercise [54], and with performance of resistance training larger doses of protein (leucine) [11] would have to be consumed. The higher protein/leucine needed to stimulate MPS in the elderly would be obtained at lower protein doses with higher quality proteins such as whey (Fig. 2, Table 1), which may be advantageous from both an energy intake and potential appetite suppression standpoint.

### Whey protein supplementation and hypertrophy with resistance training

A recent systematic review and meta-analysis of studies involving the high leucine-containing protein whey has been performed [16]. In this review the authors found that of 14 studies included in the analysis 5 were whey protein replacement studies (i.e., the whey protein was not supplemental) and 9 were supplementation studies [16]. The authors found, “…a statistically significant increase in LBM [lean body mass] (WGMD [weighted group mean difference]: 2.24 kg, 95 % CI, 0.66, 3.81) among studies that included a resistance exercise component along with WP provision.” The same authors [16] concluded, “…[the findings] support the use of WP [whey protein], either as a supplement combined with resistance exercise or as part of a weight loss or weight maintenance diet, to improve body composition parameters.” It needs to be emphasized that in this same review [16] the authors pointed out that, “…the effects of WP [whey protein] were more favorable when compared with carbohydrates than protein sources other than whey, although findings did not reach statistical significance.” This is to be expected since carbohydrates provide only energy, not amino acids, and results only in hyperinsulinemia and thus cannot stimulate a net positive protein balance [55, 56]. Nonetheless, when considered together the studies in which whey was compared to other proteins [57–62] did not show marked differences. When compared to soy, a lower quality protein (Table 1), whey protein did not show a greater effect [57, 58]. However, in only one study was resistance exercise included [57], but this study had a very small sample size. Thus, the data are limited and a firm conclusion is hard to form regarding an advantage to supplementation with whey protein over other protein sources. Since the publication of this meta-analysis [16] there have been studies published in which whey protein has been compared to soy protein [15], pea protein [63], and rice protein [64].

Volek et al. [15] conducted a long-term training study (9mo) in which a whey protein supplement was shown to significantly enhance gains in lean body mass over those seen in a soy protein-supplemented group by ~83 %. This study [15] is one of the longest protein supplementation with resistance exercise trial and highlighted the importance of protein quality in determining exercise-induced muscle mass gains. Given the importance of leucine in triggering MPS, the findings of a greater muscle mass gain in a whey supplemented group are consistent with the leucine trigger thesis (Fig. 1) for stimulation of MPS to promote hypertrophy. Importantly, the soy supplemented group had a muscle mass gain that was no different from the carbohydrate group, which is a finding that implies soy was no better than energy in the form of carbohydrate. While not whey protein per se, the findings of Hartman et al. [5] are aligned with those of Volek et al. [15]. In this study [5] bovine skimmed milk was compared to a soy protein-containing beverage and it was found that the milk drinkers gained more muscle mass than did the soy beverage consumers and a control carbohydrate only consuming group.

Joy et al. [64] studied the influence of only 8weeks of resistance training in groups of young men consuming either 48 g/d of whey or the same quantity of rice-derived protein (Oryzatein™ rice protein, Axiom Foods; see Table 1). When compared on a weight and a digestibility basis whey protein isolate has a much greater leucine content and availability (Table 1), but the researchers overcame this difference by feeding their subjects a very large quantity of protein [64]. In feeding their subjects 48 g of whey isolate and 48 g of rice protein concentrate they delivered doses of ~5.5 g and ~3.8 g of leucine, which would have hit the highest level of leucine (Fig. 1) and saturated the MPS response for both group [65]. Thus, given the saturable dose-response nature of MPS [52, 65, 66], and the subsequent hypertrophy, the results from this study [64] are not surprising. By halving the doses that these authors used [64], which arguably represents a more realistic dose of protein, then the whey protein dose would still be sufficient to maximally stimulate MPS whereas the rice protein dose would not [52, 65, 66]. Thus ‘equivalency’ of protein in this study was not a function of the protein quality itself, but of the large per-dose quantities of protein (leucine) consumed. Clearly these doses of protein were sufficient to maximally stimulate MPS in the case of both the rice and the whey supplement.

**Fig. 3:**
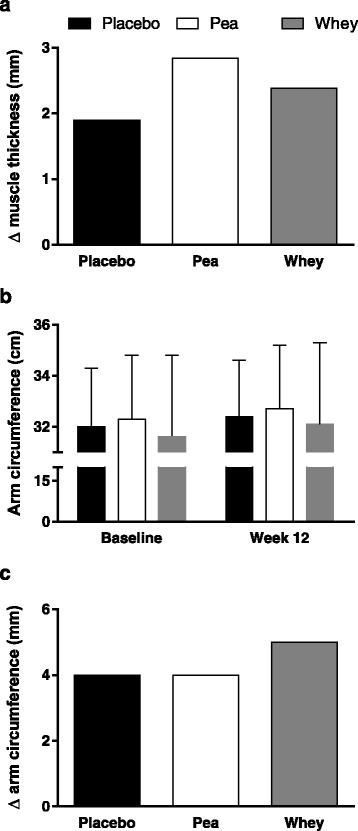
Reanalysis of data from Babault et al. [63], showing changes in muscle thickness in all groups (**a**) with data estimated from mean changes. Arm circumferences (**b**) from the same study. And the changes, estimated from means, of the change in arm circumference of the three groups (**c**)

Babault and colleagues [63] conducted a study in young men consuming either a placebo, pea protein, or whey protein. This study was a large-scale trial in which 137 subjects were included for analysis (*n* = 47, 46, and 44, respectively for the placebo, pea protein, and whey protein concentrate supplemented group). It could not be determined in this study whether the protein was truly supplemental due to a lack of reporting dietary intake data. The extra protein provided would have delivered, on a per dose basis, 1.6 g of leucine in the pea protein and 2.1 g of leucine in the whey group (both consumed twice daily) [63]. From all we know at present [41, 48, 49, 65], both of these doses were likely below an optimal leucine dose to stimulate MPS. Subjects did not engage in a full resistance exercise training program but instead performed only three resistance exercises: arm curl, lateral pulldown, and bench press, which they engaged in three times weekly for 12 weeks [63]. The results measured as biceps brachii muscle thickness showed all groups increased the thickness of their muscle (muscle thickness served here as a proxy for muscle hypertrophy), and the extent of the increase in muscle thickness was labelled by the authors as ‘trending’ to be different in the pea protein versus the whey protein supplemented group [63]. Fig. 3a shows the changes in mean muscle thickness from week 0 to week 12 in all groups. Interestingly, the increase in muscle thickness was accompanied by similar increases in strength in all groups. Thus, if as the authors suggested pea protein induced a ‘greater’ increase in muscle thickness/hypertrophy then the quality of the muscle (as force per cross-sectional area) gained would not be as great in the pea protein supplemented group than the placebo or whey protein supplemented groups. It is also hard, based on reported data from this study [63], to ascertain the significance of what the changes in muscle thickness meant in terms of true hypertrophy. Highlighting this fact, the arm circumferences reported by these authors are shown in Fig. 3b and the estimated change, based on means, are shown in Fig. 3c. While admitting the arm circumference is a crude measure of muscle hypertrophy it does, when comparing Fig. 3a, b, and c call into question what a difference in muscle thickness of less than 1 mm between the whey and pea protein supplemented groups actually means in terms of hypertrophy. In future studies, rather than proxy measures (i.e., muscle thickness), an actual measurement of hypertrophy using MRI, muscle fibre CSA, or the use of CT would be beneficial. The authors of this study [63] also conducted a ‘sensitivity’ analysis by analyzing only the changes in muscle thickness for those participants who had initial arm curl maximal strength less than the median of 25 kg. One issue with this ‘sensitivity’ analysis is that by partitioning subjects by strength lead to groups that had *n* = 17 for pea (36 % of the total sample for that group), *n* = 31 for whey (67 % of the total sample for that group), and *n* = 20 for placebo (45 % of the total sample for that group) participants for the pea, whey, and placebo groups, respectively. This analysis highlights the fact that weaker subjects were disproportionately assigned to the whey group and that a ‘sensitivity’ analysis along strength created imbalanced group sizes and a situation for a type 2 statistical error. The authors stated that, “Thickness increases between D0 and D84 were +20.2 ± 12.3 %, +15.6 ± 13.5 %, and +8.6 ± 7.3 % for Pea, Whey and Placebo, respectively. A Scheffé test showed a statistically significant difference between Pea and Placebo [2.51 mm] whereas there was no significant difference between Whey and Pea [1.21 mm]… nor between Whey and Placebo [1.29 mm].” (p. 5) [63]. A number of the findings and claims that the results of this study [63] were due to pea protein are difficult to reconcile with the leucine content and availability of the pea versus the whey protein (Table 1 and Fig. 2) and known relationships with leucine and MPS [52, 65, 66]. Moreover, the ‘sensitivity’ analysis used by these authors created an imbalanced group assignment that lead to the creation of disproportionate group sizes and, it is proposed, a spurious conclusion regarding the efficacy of pea versus whey protein.

### Protein blends

Blended proteins (mixtures of different isolated protein sources) have been sparingly studied, but in all cases where MPS has been measured blends of proteins give similar responses to leucine-matched whey protein [67, 68]. A theory behind why blends of proteins might be considered to be more effective than isolated proteins would be that the delivery of amino acids could be extended or that certain amino acids are enriched in some sources [69]; however, as the main driver of MPS the most relevant amino acid is leucine. For example, Reidy et al. compared a blend of whey (25 % by weight), caseinate (50 % by weight), and soy (25 % by weight) to a leucine-matched quantity of whey. There were differences in the aminoacidemia obtained following ingestion of these two treatments that were accompanied by different time courses of muscle protein synthesis, however, on balance the incorporation of amino acids into muscle protein was not different between treatments. These findings are in agreement with the leucine trigger concept (Fig. 1) and highlight the importance of leucine as an amino acid that needs to be considered in comparing supplemental sources of protein. Future research on blends of protein will need to focus on comparing protein blends to other protein sources in longer-term studies with hypertrophy and/or strength as a main outcome.

### Other supplemental proteins

A number of other supplemental protein sources have now become available to consumers including hemp protein and ‘insect-based’ protein, and other plant-derived proteins. While it is not possible to make specific comparisons to the proteins examined here, the leucine content and quality (due to processing and the presence of anti-nutritional compounds [70] unless they are removed) will be lower than most if not all more commonly available supplemental proteins (Table 1). For example, the PDCAAS (DIAAS is not available) scores were estimated by House et al. [71] to be 0.49–0.53 for whole hemp seed, 0.46–0.51 for hemp seed meal, 0.63–0.66 for dehulled hemp seed. Obviously, the hull of the seed contained a high quantity of antinutritional factors and its removal improved protein quality. Lysine was the first limiting amino acid in all hemp-based proteins [71]. It is important that these proteins now be assessed and their DIAAS scores estimated so that we can make a true assessment of their quality when compared to more commonly-used isolated proteins.

Collagen has also gained in popularity as a source of protein in supplemental sources. This is intriguing given the PDCAAS score of collagen is zero due to the fact that it is lacking in tryptophan. However, protein blends containing collagen, which even with added tryptophan would have a PDCAAS score of 0.39, would add mostly dispensable amino acids (and admittedly protein content on the product label) to existing protein content and would not improve protein quality. In addition to collagen a variety of insect-derived proteins have been assessed for their protein quality and, not unsurprisingly, there are wide variation in protein digestibility [72]. Nonetheless, insect protein tends, in general, to be lower quality and lower in IAA than comparable proteins we have examined here. Most often, in protein sources claiming to be insect protein, that are available for human consumption, they are not pure insect protein but instead blends of insect and other proteins such as a rice, hemp, and/or soy protein. Thus, the protein quality of such protein blends is a function not of the insects and unlikely to be as high as most common supplemental proteins (Table 1). As a result, to achieve a dose of leucine sufficient to stimulate MPS to an appreciable degree unless larger quantities (40–50 g per serve) of the protein are consumed; thus, these protein sources would be inferior to those proteins described in Table 1. It is important that future studies in this area emphasize that discussion of protein ‘quality’ using at least the content of IAA, PDCAAS (if available), or DIAAS (if possible) to accurately characterize proteins for use in human clinical research.

### Recommendation and future directions

Future studies in which protein is given to participants to assess the impact of this macronutrient on resistance training-induced hypertrophy need to consider the following:

1. Is the protein given a supplement? A supplemental protein, by definition, is in addition to the persons’ normal dietary intake. Thus, researchers are urged to make some effort to assess normal dietary intake prior to the supplementation and exercise intervention.
2. Is the allocation of the protein supplements blinded and is subject compliance assessed? While blinding of the subjects and investigators to the supplementation is self-explanatory, objective measures of compliance are rarely used. As one biomarker of compliance with an increased protein intake urinary or serum urea levels could be assessed.
3. Is the study of sufficient duration and is adequately powered to detect differences? While the time course of muscle hypertrophy is not known exactly an 8weeks intervention would be considered the minimum as true hypertrophy (i.e., measured with muscle biopsies or by MRI and/or CT) is detectable/measurable after 6–8 weeks of resistance training [73–76]. Study power is often not mentioned, but it should be and the minimum significant effect as well as the degree of change in hypertrophy needs to be outlined. To detect hypertrophy differences in proteins of differing protein quality it appears that this should be in the range of at least 25 subjects per group [5, 15] and for a period of at least 10–12 weeks in novice lifters and possibly longer in experienced lifters.
4. CONSORT (http://www.consort-statement.org/) guidelines need to be followed. The CONSORT guidelines provide a minimum set of recommendations for reporting of randomized trials. Adherence to this standard allows an easy cross-comparison of one trial to the next. This aids in standardization, complete and transparent reporting, and aids in interpretation.
5. An appropriate placebo needs to be used. Comparisons of protein to carbohydrate (i.e., to try and make the interventions isoenergetic, but not isonitrogenous) are more likely to show an effect of supplementation. Some studies have compared protein sources based on leucine content [67], with the expected outcome that protein turnover is no different between the two, however, it would seem to make more sense to compare protein sources and doses that are isonitrogenous.

A further consideration in recommendations for using lower quality proteins is their potential use in populations such as the elderly for whom a requirement for protein [77, 78], and more importantly leucine [42, 79, 80], for retention of muscle mass appears to be higher than that of younger persons. For older persons lower quality protein sources would have to be rigorously tested. This sentiment would be particularly true in older persons with marginal energy intake and or lower levels of physical activity for whom high quality (and nutrient-dense) sources of protein would be recommended [54, 81, 82].

## Conclusions

Protein quality appears to play a role in determining resistance exercise-induced muscle hypertrophy; however, the effect is more difficult to detect compared to a comparison between the protein and an isoenergetic source of carbohydrate. The leucine content of a protein is the strongest determinant of the capacity of a protein to affect MPS and likely hypertrophy. While the prior performance of exercise will lower the threshold for protein/leucine required to stimulate MPS the importance of leucine content for MPS and likely subsequent hypertrophy needs to be appreciated in the context of not only its content in a protein source but also its digestibility. While there are few studies that have actually derived the DIAAS of proteins this variable is something that needs to be considered moving forward. When comparing proteins of differing quality, larger adequately powered rigorous trials need to be run to assess the impact of protein quality in determining resistance exercise-induced hypertrophy. Future studies in this area may wish to consider the recommendations outlined here in terms of trying to improve overall study quality and, importantly, to allow for easier comparisons between trials.

## Abbreviations

AA: Amino acid
CT: Computed tomography
DIAAS: Digestible indispensable amino acid score
IAA: Indispensable amino acid
LBM: Lean body mass
MPB: Muscle protein breakdown
MPS: Muscle protein synthesis
MRI: Magnetic resonance image
mTORC1: Mechanistic target of rapamycin complex-1
PDCAAS: Protein digestibility-corrected amino acid score
WGMD: Weighted group mean difference
WP: Whey protein
